# Individual Morphological Brain Network Construction Based on Multivariate Euclidean Distances Between Brain Regions

**DOI:** 10.3389/fnhum.2018.00204

**Published:** 2018-05-25

**Authors:** Kaixin Yu, Xuetong Wang, Qiongling Li, Xiaohui Zhang, Xinwei Li, Shuyu Li

**Affiliations:** ^1^School of Biological Science & Medical Engineering, Beihang University, Beijing, China; ^2^Beijing Advanced Innovation Centre for Biomedical Engineering, Beihang University, Beijing, China

**Keywords:** individual morphological brain network, multivariate Euclidean distance, mild cognitive impairment, multiple morphological features, classification

## Abstract

Morphological brain network plays a key role in investigating abnormalities in neurological diseases such as mild cognitive impairment (MCI) and Alzheimer's disease (AD). However, most of the morphological brain network construction methods only considered a single morphological feature. Each type of morphological feature has specific neurological and genetic underpinnings. A combination of morphological features has been proven to have better diagnostic performance compared with a single feature, which suggests that an individual morphological brain network based on multiple morphological features would be beneficial in disease diagnosis. Here, we proposed a novel method to construct individual morphological brain networks for two datasets by calculating the exponential function of multivariate Euclidean distance as the evaluation of similarity between two regions. The first dataset included 24 healthy subjects who were scanned twice within a 3-month period. The topological properties of these brain networks were analyzed and compared with previous studies that used different methods and modalities. Small world property was observed in all of the subjects, and the high reproducibility indicated the robustness of our method. The second dataset included 170 patients with MCI (86 stable MCI and 84 progressive MCI cases) and 169 normal controls (NC). The edge features extracted from the individual morphological brain networks were used to distinguish MCI from NC and separate MCI subgroups (progressive vs. stable) through the support vector machine in order to validate our method. The results showed that our method achieved an accuracy of 79.65% (MCI vs. NC) and 70.59% (stable MCI vs. progressive MCI) in a one-dimension situation. In a multiple-dimension situation, our method improved the classification performance with an accuracy of 80.53% (MCI vs. NC) and 77.06% (stable MCI vs. progressive MCI) compared with the method using a single feature. The results indicated that our method could effectively construct an individual morphological brain network based on multiple morphological features and could accurately discriminate MCI from NC and stable MCI from progressive MCI, and may provide a valuable tool for the investigation of individual morphological brain networks.

## Introduction

Morphological brain network refers to the intracortical similarities in gray matter morphology (He et al., 2007) which plays a key role in investigating brain abnormalities in neurological diseases. By analyzing morphological brain network features, the abnormalities in connectivity parameters can be found in patients (Yao et al., 2010; Tijms et al., 2013). More importantly, sensitive biomarkers for clinical diagnosis can be detected in brain networks from cases of Alzheimer's disease (He et al., 2008, 2009), schizophrenia (Bassett et al., 2008; Zhang et al., 2012) and epilepsy (Bernhardt et al., 2008, 2009).

Although previous morphological brain network studies achieved significant breakthroughs, they largely depended on group-level anatomical correlations of cortical morphology (He et al., 2007; Zhang et al., 2012). For example, He et al. (2007) constructed a network for each group by quantifying morphological relations characterized by the Pearson correlation coefficient between averaged regional morphological measures among participants. However, this method only works with a relatively large number of participants (Kong et al., 2014). In addition, it remains unclear if there are changes in brain networks at the individual level (Saggar et al., 2015). Therefore, it is necessary to construct morphological brain networks at the individual level for the direct analysis of individual differences.

Recently, several methods have been proposed to construct individual morphological brain networks either using a single feature or multiple morphological features. By using gray matter volume as the morphological measure, Tijms et al. (2012) proposed an individual morphological brain network by computing the correlation between two 27-voxel sets from two rigid cubes. There were some studies constructing individual brain networks by averaging the vertex value (e.g., cortical thickness) within regions of interest (ROI) (Dai et al., 2013; Wee et al., 2013; Kim et al., 2016) or by estimating interregional similarity in the distribution of regional morphological measures (e.g., cortical thickness or volume) (Kong et al., 2014; Zheng et al., 2015). Wang et al. (2016) employed graph-based analyses to support individual morphological network analysis as a meaningful and reliable method when characterizing brain structural organization. Some recent studies (Li et al., 2017; Seidlitz et al., 2017) built individual morphological networks with multiple morphological features extracted from the cortical surface. Each type of morphological feature has specific neurological and genetic underpinnings. Volumetric measures (i.e., cortical thickness, gray matter volume) reflect the size, density and arrangement of cells (neurons, neuroglia, and nerve fibers) (Parent and Carpenter, 1996) and surface area is linked to the number of mini columns in the cortical layer (Rakic, 1988). Geometric measures (i.e., sulcal depth, curvature, and metric distortion) mainly reflect the cortical folding pattern (Van Essen, 1997; Cachia et al., 2003; Lohmann et al., 2008). Li et al. (2014) found that various morphological features had unique contributions to the classification of the amnestic MCI (aMCI) and NC. In the two studies (Li et al., 2017; Seidlitz et al., 2017), a morphological feature vector was used to represent one region and pairwise inter-regional Pearson correlations were used to construct brain network, while not considering the distribution of the intra-regional morphological features.

In this paper, we proposed a novel individual morphological brain network method by defining multivariate Euclidean distance to describe the inter-regional similarity based on multiple morphological features. First, multivariate Euclidean distance was calculated by using the six morphological features of all of the vertices within each region. Second, the Min-Max normalization for Euclidean distance was performed to minimize possible bias in different ranges of different subjects. Finally, the normalized Euclidean distance was converted to a similarity measurement using an exponential function. Then, we validated the proposed method by computing the topological properties of individual brain networks, i.e., small-world, hubs and intraclass correlation coefficient (ICC) in 24 healthy subjects. In addition, we applied the edges of each individual morphological network as features to discriminate the MCI and NC in the AD Neuroimaging Initiative (ADNI) dataset. The accuracy of classification was used to assess the effectiveness of our method.

## Materials and methods

### Participants

The first dataset used in this study consisted of 24 right-handed healthy subjects (12 men with ages ranging from 25 to 29 years with mean = 27.17 years, and standard deviation = 1.40; 12 women with ages ranging from 26 to 30 years with mean = 27.83 years, and standard deviation = 1.11). All subjects were native Chinese speakers who had grown up in China. All subjects provided written informed consent; in addition, the local ethics committee approved this study.

The subjects were scanned twice within a 3-month period. All of the MRI data were obtained using a SIEMENS Trio Tim 3.0T scanner with a 12-channel phased array head coil in the Imaging Center for Brain Research, Beijing Normal University. The brain structural images were acquired using T1-weighted, sagittal 3D magnetization prepared rapid gradient echo (MPRAGE) sequences. The sequence parameters had a repetition time (TR) = 2,530 ms, echo time (TE) = 3.39 ms, inversion time (TI) = 1,100 ms, flip angle = 7°, FOV = 256 ^*^ 256 mm, in-plane resolution = 256 ^*^ 256, slice thickness = 1.33 mm, and 144 sagittal slices covering the whole brain.

The second dataset used in this study was obtained from the ADNI database (adni.loni.usc.edu). The ADNI was launched in 2003 as a public-private partnership, led by Principal Investigator Michael W. Weiner, MD. The primary goal of ADNI has been to test whether serial MRI, positron emission tomography (PET), other biological markers, and clinical and neuropsychological assessment can be combined to measure the progression of MCI and early Alzheimer's disease (AD). This study was carried out in accordance with the recommendations of the ADNI database with written informed consent from all subjects. The protocol was approved by the ADNI coordinating committee.

The eligibility criteria for inclusion of subjects are described at http://adni.loni.usc.edu/wp-content/uploads/2010/09/ADNI_GeneralProceduresManual.pdf. General criteria for MCI were as follows: (1) Mini-Mental-State-Examination (MMSE) scores between 24 and 30 (inclusive), (2) a memory complaint, objective memory loss measured by education adjusted scores on the Wechsler Memory Scale Logical Memory II, (3) a Clinical Dementia Rating (CDR) ≥ 0.5, and (4) absence of significant levels of impairment in other cognitive domains, essentially preserved activities of daily living, and an absence of dementia.

Three hundred and thirty-nine subjects, which included 170 MCI patients and 169 NC subjects were analyzed in this study. Age, gender and education in the MCI group were matched with the NC group. All subjects received the baseline clinical/cognitive examinations including 1.5T structural MRI scan and were reevaluated at specified intervals (6 or 12 months). The baseline scans were used in our experiments. The 170 MCI subjects included two subcategories: 86 stable MCI (sMCI) and 84 progressive MCI (pMCI). Subjects who converted to AD within 24 months were classified as pMCI, and those not converting into AD within the same period were classified as sMCI. The 169 NC subjects were not converted to MCI or AD within 24 months. The demographic information and clinical characteristics of the participants involved in this study are shown in Table 1.

**Table 1:** Subject demographic and clinical characteristics.

	**MCI (*n* = 170)**	**sMCI (*n* = 86)**	**pMCI (*n* = 84)**	**Control (*n* = 169)**
Gender (M/F)	104/66	53/33	51/33	88/81
Age	74.8 ± 6.7	74.6 ± 6.4	75.1 ± 7.2	75.7 ± 5.1
Education	15.7 ± 3.0	15.8 ± 3.1	15.7 ± 3.0	16.0 ± 2.7
MMSE	26.9 ± 1.7	27.4 ± 1.8	26.4 ± 1.7	29.1 ± 0.9
CDR	1.6 ± 0.8	1.5 ± 0.7	1.8 ± 1.0	0 ± 0.1

*Age, education, MMSE and CDR are expressed as the mean ± SD. There were no significant differences between the MCI and the control group and between the sMCI and pMCI group in gender, age and education years. The MCI with control groups, and sMCI with pMCI group showed significant differences in the MMSE and CDR. MCI, mild cognitive impairment; sMCI, stable mild cognitive impairment; pMCI, progressive mild cognitive impairment; M/F, Male/Female; MMSE, Mini-Mental-State-Examination; CDR, Clinical Dementia Rating*.

### Image processing

The same pre-processing pipeline was applied in the two datasets by using the FreeSurfer image analysis suite v4.3 (http://surfer.nmr.mgh.harvard.edu/). For the second dataset, the pre-processed images were downloaded from the public ADNI site. The pipeline for T1-weighted scans contained (1) registration to the Talairach space, (2) correction for intensity bias, (3) skull stripped from the intensity normalized image, (4) segmentation into white matter, gray matter or cerebrospinal fluid, (5) cutting planes to sphere the hemispheres and remove the cerebellum and brain stem, (6) generation of a single connected mass representing the white matter structure of each hemisphere, and (7) surface tessellation, refinement, and deformation for each hemisphere (Dale et al., 1999). A variety of morphological features such as volumetric (cortical thickness, surface area, and gray matter volume) and geometric (sulcal depth, metric distortion, and mean curvature) measures at each vertex on the pial surface were extracted after the preprocessing. Then, the surface data were resampled to a common subject (usually an average subject) and smoothed with a Gaussian filter (FWHM = 5 mm).

### Construction of individual morphological brain network

A brain network is typically defined as *G* = *(V, E)*, where *V* denotes the set of nodes (or vertices) and *E* denotes the set of edges (or links). In this paper, we parceled the cortical cortex into 68 cortical ROIs based on the Desikan-Killiany Atlas (Desikan et al., 2006). Here, we assumed that nodes represent cortical regions and edges represent the similarity of two cortical regions. Each individual network shares the same set of 68 nodes, which facilitates the comparisons using the edges. Dissimilarity connectivity is measured by the formula below (Székely and Rizzo, 2004). Let *A* and *B* denote the ROIs of the *k*th subject, and then the combined Euclidean distance *e*_*k*_(*A, B*) is defined as:

(1) $$\begin{array}{l} e_{k}(A,B)=\frac{n_{1}n_{2}}{n_{1}+n_{2}}(\frac{2}{n_{1}n_{2}}\sum_{i=1}^{n_{1}}\sum_{j=1}^{n_{2}}{\left\|a_{i}-b_{j}\right\|}_{2}\\ \;-\;\frac{1}{n_{1}^{2}}\sum_{i=1}^{n_{1}}\sum_{j=1}^{n_{1}}{\left\|a_{i}-a_{j}\right\|}_{2}-\frac{1}{n_{2}^{2}}\sum_{i=1}^{n_{2}}\sum_{j=1}^{n_{2}}{\left\|b_{i}-b_{j}\right\|}_{2}) \end{array}$$

Let *A* = {**a**_1_, …, **a**_*n*_1__} and *B* = {**b**_1_, …, **b**_*n*_2__}, where ***a*** and ***b*** denote vertices in *A* and *B*, respectively. These elements represent morphological features, which could be either one-dimensional or multi-dimensional. *n*_1_ and *n*_2_ are the numbers of vertices in *A* and *B*. Euclidean distance is computed by the 2-norm (||.||_2_). The first part of the formula $\frac{2}{n_{1}n_{2}}\overset{n_{1}}{\underset{i=1}{\sum }}\overset{n_{2}}{\underset{j=1}{\sum }}{\left||{\text{a}}_{i}-{\text{b}}_{j}|\right|}_{2}$ describes the Euclidean distance for any pair of vertices between *A* and *B*. $\frac{1}{n_{1}^{2}}\overset{n_{1}}{\underset{i=1}{\sum }}\overset{n_{1}}{\underset{j=1}{\sum }}{\left||{\text{a}}_{i}-{\text{a}}_{j}|\right|}_{2}$ and $\frac{1}{n_{2}^{2}}\overset{n_{2}}{\underset{i=1}{\sum }}\overset{n_{2}}{\underset{j=1}{\sum }}{\left||{\text{b}}_{i}-{\text{b}}_{j}|\right|}_{2}$ are the Euclidean distances for any pair of vertices within *A* and *B*, respectively.

A smaller intra-regional Euclidean distance indicating uniform morphological feature distribution within ROI results in a distance *e*(*A, B*) is more dependent on the Euclidean distance between pairs of vertices in *A* and *B*. Moreover, the distance *e*(*A, B*) will be influenced if the morphological feature distribution within the ROI is unequal. When *A* and *B* have the same morphological feature distribution, the combined Euclidean distance *e*(*A, B*) = 0.

After calculation of the combined Euclidean distance matrix that reflected the dissimilarity between brain regions, Min-Max normalization was proposed to minimize possible bias in different ranges of different subjects. We chose the Min-Max normalization because of its boundness and direct reflection of the dissimilarity. The Min-Max normalization between regions *A* and *B* of the *k*th subject is computed as:

(2) $$e_{k}\_n(A,B)=\frac{e_{k}(A,B)-e_{k\_min}}{e_{k\_max}-e_{k\_min}}$$

where *e*_*k*_min_ and *e*_*k*_max_ are the minimum and maximal value in the dissimilarity connectivity of the *k*th subject, respectively. The value of *e*_*k*__*n*(*A, B*) can be converted to a similarity measurement using the following equation:

(3) $$c_{k}(A,B)=\exp(-e_{k}\_n(A,B))$$

Based on the above calculation, a 68^*^68 diagonal symmetry correlation matrix of each subject was obtained. The *c*_*k*_(*A, B*) ranges from 0 to 1, and 1 represents that the two morphological feature distributions are identical.

### Method validation

We validated the above method by computing the topological properties of the individual brain network, i.e., small-world, hubs and intraclass correlation coefficient (ICC) in the first dataset. In addition, we applied the edges of each individual morphological network as features to discriminate the MCI and NC in the ADNI dataset. The accuracy of classification was used to assess the effectiveness of our method.

#### Topological properties of networks

We constructed the individual morphological brain network based on the proposed method in a six-dimension situation in the first dataset. The small-world configurations, hubs and reproducibility of individual brain network were calculated and analyzed. The network properties were computed using the Graph-theoretical Network Analysis (GRETNA) toolkit (Wang et al., 2015).

For small-world configurations, the clustering coefficient (*Cp*), minimum path length (*Lp*), γ, λ and σ were calculated. Small-worldness (Watts and Strogatz, 1998; Humphries et al., 2006) can be demonstrated mathematically as:

$$\gamma =\frac{Cp}{Cp^{random}}>1,\lambda =\frac{Lp}{Lp^{random}}\approx 1\text{ and }\sigma =\frac{\gamma }{\lambda }>1$$

where *random* represents a random network that consists of the same number of nodes and edges.

The betweenness centrality (BC) is defined as the number of shortest paths between any two nodes running through the given node (Freeman, 1977) and measures the nodal ability of information flow throughout the network. The hubs were defined as the nodes that achieved a higher BC than the sum of the mean and standard deviation for the entire network.

The intraclass correlation coefficient (ICC) was used to estimate the reproducibility of the topological properties of the network (Shrout and Fleiss, 1979). ICC was defined as the fraction of the variance of the chosen graphic property between subjects to the total variance, which is the summation variance of between and within subjects of that property:

(4) $$ICC=\frac{\sigma_{between}^{2}}{\sigma_{between}^{2}+\sigma_{within}^{2}}$$

If the measurements of repeated scans are consistent for each subject, the ICC would be close to one. An ICC value above 0.75 is considered excellent, and one ranging from 0.59 to 0.75 is considered good (Cicchetti and Sparrow, 1981).

#### Classification between MCI and NC groups

For the second dataset, we used the support vector machine (SVM) classifiers with leave-one-out cross validation (LOOCV) to test the effectiveness of our method. Additionally, feature selection is employed for each individual morphological brain network before classification regarding the curse of dimensionality.

##### Feature selection

Each network has *p* = *V* × (*V* − 1)/2 = 2278 edges. Due to the high dimensionality of the network features and a small number of samples, also namely, the curse of dimensionality, the classification model often confronts problems such as overfitting and under generalization. Feature selection is considered to reduce the irrelevant or redundant features and improve the performance of classifiers. The least absolute shrinkage and selection operator (Lasso) (Tibshirani, 1996) was applied for feature selection.

Specifically, Lasso was put forward by Tibshirani (1996) for parameter estimation and feature selection in regression analysis. The Lasso algorithm does not focus on selection of subsets but rather on defining a continuous shrinking operation that can produce coefficients of redundant components to zero. It has been shown in the literature (Yamada et al., 2012; Kamkar et al., 2015) that the algorithm can effectively select the relevant features in high dimensional data space. Sparse linear regression is applied for Lasso features calculation with L_1_-norm regularization. In the training set, let matrix $X={\left[x_{1},x_{2},\ldots ,x_{n}\right]}^{T}\in {\mathbb{R}}^{n\times m}$ represent *m* features of *n* subjects, $y={\left[y_{1},y_{2},\ldots y_{i},\ldots y_{n}\right]}^{T}\in {\mathbb{R}}^{n\times 1}$ be an *n* dimension corresponding to sample labels (*y*_*i*_ = 1 for MCI and *y*_*i*_ = -1 for NC) and *m* denotes the number of edges except the duplicated part in the individual brain network. The linear regression model is defined as follows:

(5) $$\overset{\wedge }{y}=Xw$$

where $w={\left[w_{1},w_{2},\ldots w_{n}\right]}^{T}\in {\mathbb{R}}^{n\times 1}$ denotes the regression coefficient vector and $\overset{\wedge }{y}$ denotes the predicted label vector. The objective function is minimized as follows to estimate *w*:

(6) $$\underset{w}{\min}\frac{1}{2}{\left\|Xw-y\right\|}_{2}^{2}+\lambda {\left\|w\right\|}_{1}$$

where λ > 0 is a regularization parameter in control of the sparsity of the model, i.e., many entries of *w* are zeros. ||*w*||_1_ is the L_1_-norm of *w* defined as $\sum_{i=1}^{n}|w_{i}|$. The SLEP package (Liu et al., 2009) was used for solving sparse linear regression. If an edge is selected as a feature in each iteration of the LOOCV classification, the edge is considered as discriminative in the brain network.

##### Classification

**Figure 1:**
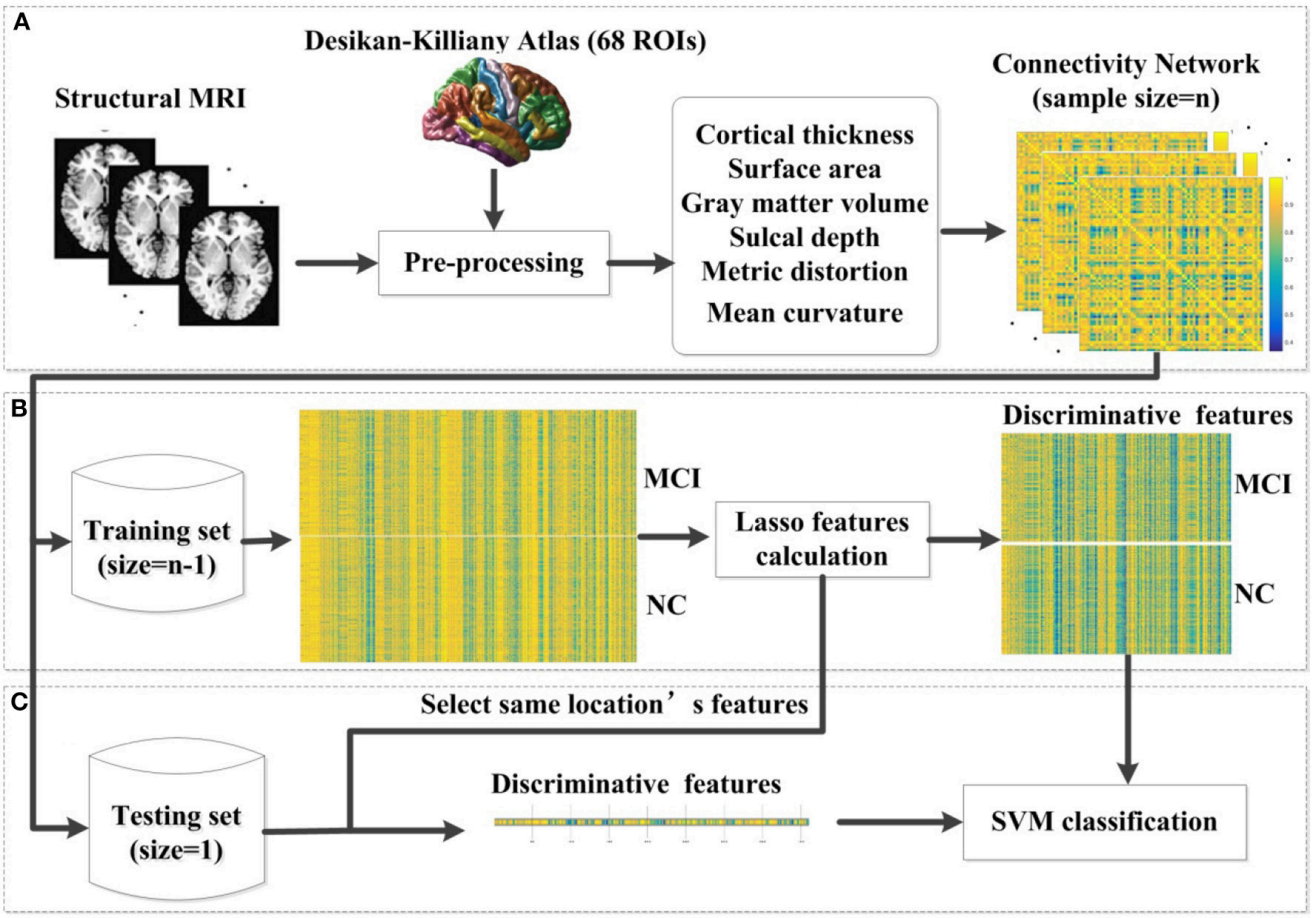
The proposed classification framework. **(A)** The step of pre-processing was accomplished using FreeSurfer. For each vertex in each region, six morphological features were extracted. After the pre-processing, we constructed an individual brain network based on the multivariate Euclidean distance. **(B)** In each LOOCV classification, we first constructed the combination of the training set, and then applied the lasso features calculation for feature selection. **(C)** We selected the same location's features in the testing set. Then the SVM classifier was implemented for classification. MCI, mild cognitive impairment; NC, normal controls; SVM, support vector machine; LOOCV, leave-one-out cross validation.

According to the selected features described above, a commonly used classifier SVM was implemented using the LIBSVM library (Chang and Lin, 2011) in MATLAB, with a radial basis function (RBF) kernel and an optimal value for the penalized coefficient *C* (a constant determining the tradeoff between training error and model flatness). The RBF kernel was utilized for its good performance especially on small sample problems (Hertz et al., 2006) and defined as follows:

(7) $$K(x_{1},x_{2})=\exp(-\frac{{\left\|x_{1}-x_{2}\right\|}^{2}}{2\sigma^{2}})$$

where *x*_1_ and *x*_2_ are two feature vectors and σ is the width of the Gaussian kernel. To obtain the optimal SVM model, we selected the optimal hyperparameters (C and σ) through a grid-search. Specifically, the classification was performed via a LOOCV in which one subject was selected as the testing set and the rest were used as the training set. The parameters were changed after all samples were classified to estimate the LOOCV accuracy. In the end, the average accuracy across all subjects was computed as a performance measurement. The hyperparameter values that lead to the highest performance are then selected. The pipeline of our classification framework for MCI and NC is presented in Figure 1. The pipeline of classification framework for sMCI and pMCI is same as the classification framework for MCI and NC.

## Results

### Small-world configurations

**Figure 2:**
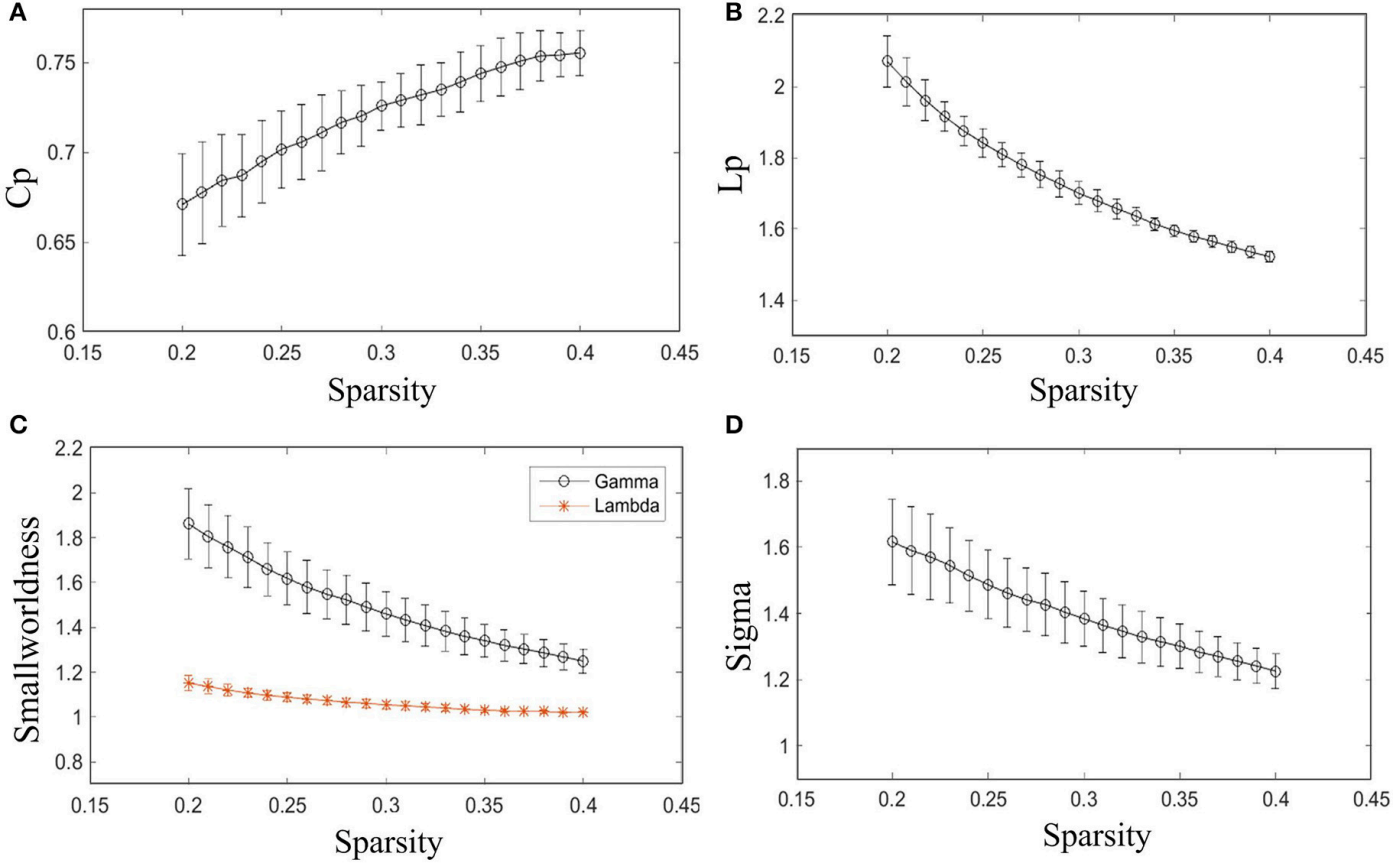
Small-world configurations of the individual morphological brain network. **(A–D)** The average *Cp, Lp*, γ, λ, and σ of the subjects for each sparsity (from 20 to 40% with a step size of 1%). The error bar indicates the standard deviation caused by different subjects.

As shown in Figure 2, γ is larger than one (max = 1.86, min = 1.25) throughout the whole sparsity range, while λ is close to one (max = 1.15, min = 1.02) by our method. Hence, the individual morphological brain networks exhibit a higher *Cp* than the random network, while maintaining a similar *Lp*. As expected, σ was found to be larger than one (max = 1.62, min = 1.23) throughout the entire sparsity range. The results showed the existence of small world property in the constructed individual morphological brain network by using six features. Moreover, as the sparsity increased, the increase of *Cp* and decrease of *Lp*, λ, σ, and γ in Figure 2 are in accordance with the variation tendency of previous reports (Kong et al., 2015; Li et al., 2017).

Furthermore, the sparsity of 23% is highlighted for convenient comparison with previous studies (Tijms et al., 2012; Kong et al., 2015). As listed in Table 2, our results are similar to previous individual-based morphological brain network studies, whereas the population-based morphological brain networks and functional networks exhibit smaller results than our method in most small world configurations.

**Table 2:** Comparison of small world configurations between the present study and previous studies.

**Method**	***N***	***Cp***	***Lp***	**γ**	**λ**	**σ**	***S (%)***
**INDIVIDUAL-BASED MORPHOLOGICAL BRAIN NETWORK**							
Our method	68	0.69	1.92	1.71	1.10	1.54	23
Li's method (Li et al., 2017)	68	0.62	2.23	1.81	1.22	1.52	23
Kong's method (Kong et al., 2015)	90	0.66	1.92	1.74	1.15	1.50	23
Tijms's method (Tijms et al., 2012)	6,982	0.53	1.86	1.35	1.05	1.28	23
**POPULATION-BASED MORPHOLOGICAL BRAIN NETWORK**							
He's method (He et al., 2007)	54	≈0.3	≈1.6	≈1.35	≈1	≈1.35	23
Yao's method (Yao et al., 2010)	90	≈0.49	≈1.89	≈1.62	≈1.1	≈1.47	23
Zhu's method (Zhu et al., 2012)	90	≈0.26	*NR*	≈1.20	≈1.03	≈1.17	23
**FUNCTIONAL BRAIN NETWORK**							
Van's method (Van Essen, 1997)	10,000	≈0.52	≈1.75	≈1.9	≈1.03	≈1.85	20
Zhang's method (Zhang et al., 2011)	90	≈0.33	≈1.65	≈1.3	≈1	≈1.4	23

*N, Cp, and Lp denote the number of nodes in the networks, the average clustering coefficient and the average shortest path length, respectively. γ represents the ratio of the clustering coefficient of the network over that of the random network. λ represents the ratio of the average shortest path length of the network over that of the random network. σ indicates the small-worldness. The small world attributes of previous studies are inferred (with ≈). NR, not reported*.

### Hubs

Hubs were investigated for all subjects and sparsities. A total of four hub regions were identified throughout the entire sparsity range across all subjects, including the left and right frontal pole, right rostral anterior cingulate and right transverse temporal cortex.

### Reproducibility

The reproducibility of our method was evaluated by measuring the ICCs of network properties for scans with acquisitions of two different time points in the same subjects. The ICC was investigated throughout the entire sparsity range. The *Cp, Lp*, and BC were examined in this study.

**Figure 3:**
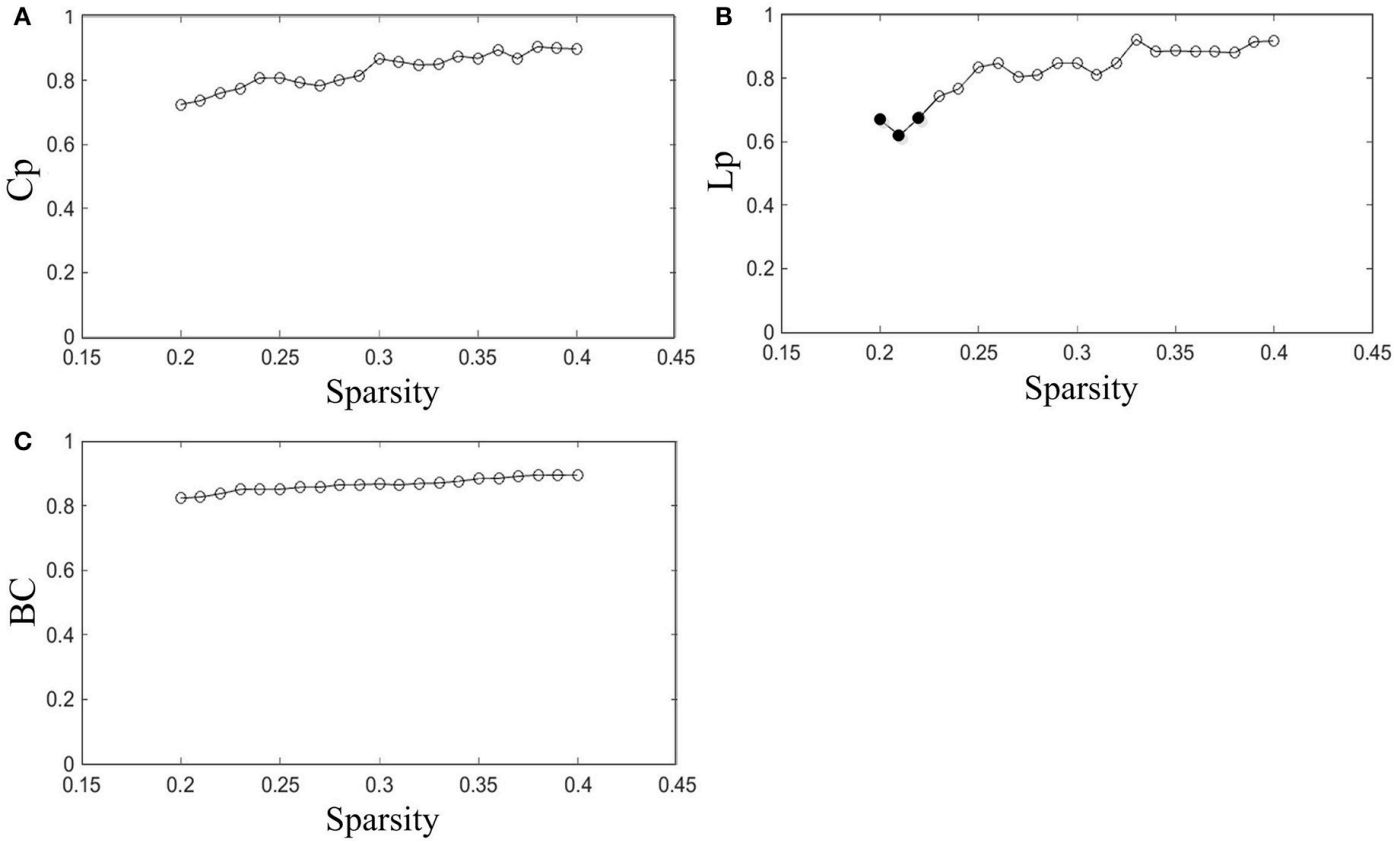
Reproducibility of the method. **(A–C)** Represent the ICC of *Cp, Lp*, and BC as a function of sparsity, respectively. The hollow dots indicate the significant results.

The results indicated that *Cp* is highly reproducible (minimum ICC = 0.72, average ICC = 0.83), as shown in Figure 3A. Moreover, the reproducibility of *Lp* (minimum ICC = 0.62, average ICC = 0.82) and BC (minimum ICC = 0.82, average ICC = 0.87) are shown in Figures 3B,C. Most results of ICC were significant, except for *Lp* at sparsity of 20, 21, and 22% (*p* = 0.098, 0.13 and 0.10, separately). The reliability of our method performed well in accordance with previous studies (Cicchetti and Sparrow, 1981; Li et al., 2017). For example, the reproducibility of *Cp* and *Lp* are similar to Li's results (minimum *Cp* ICC = 0.71, average *Cp* ICC = 0.83; minimum *Lp* ICC = 0.63, average *Lp* ICC = 0.81) and the reproducibility of BC was better than Li's result (minimum BC ICC = 0.629, average BC ICC = 0.78).

### Classification performance

In this subsection, we made a comparison of classification accuracies between our method and other methods as reported in previous studies, which included Kong's method (Kong et al., 2014), Kim's method (Kim et al., 2016), Zheng's method (Zheng et al., 2015), Dai's method (Dai et al., 2013), and Wee's method (Wee et al., 2013). The details of these methods are described in Table 3.

**Table 3:** The methods of constructing individual morphological brain networks in previous studies.

**Author**	**Methods**	**Explanation**
Kong (Kong et al., 2014)	$KL\left(i,j\right)=\int_{x}\left(i\left(x\right)\log\left(\frac{i\left(x\right)}{j\left(x\right)}\right)\;+\;j\left(x\right)\log\left(\frac{j\left(x\right)}{i\left(x\right)}\right)\right),c\left(i,j\right)=e^{-KL\left(i,j\right)}$	*i(x)* and *j(x)* denote the probability density functions (PDF) of *i* and *j* respectively.
Kim (Kim et al., 2016)	$Z\left(i,j\right)=\frac{T\left(i\right)-T\left(j\right)}{\sigma_{j}}$, $c\left(i,j\right)=\frac{\left(Z\left(i,j\right)\;+\;Z\left(j,i\right)\right)}{2}$	*T(i)* and *T(j)* denote the mean value of cortical thickness in *i* and *j* respectively, σ_*i*_ and σ_*j*_ denote the standard deviation of regional cortical thickness of regions *i* and *j*. η is an input parameter.
Wee (Wee et al., 2013)	*d*(*i, j*) = [*T*(*i*)−*T*(*j*)]^2^, $\sigma =\sqrt{\sigma_{i}\;+\;\sigma_{j}}$, $c\left(i,j\right)=\exp\left(-\frac{d\left(i,j\right)}{2\sigma^{2}}\right)$	
Dai (Dai et al., 2013)	*d*(*i, j*) = [*T*(*i*)−*T*(*j*)]^2^, $c\left(i,j\right)=\exp\left(-\frac{d\left(i,j\right)}{\eta }\right)$	
Zheng (Zheng et al., 2015)	$c_{precision}\left(i,j\right)=\frac{1}{m}\overset{m}{\underset{p=1}{\sum }}|t_{i}^{p}-T\left(i\right)|\frac{1}{n}\overset{n}{\underset{q=1}{\sum }}|t_{j}^{q}-T\left(j\right)|$, $c_{rough}\left(i,j\right)={\left|T\left(i\right)-T\left(j\right)\right|}^{2}$	*t* denotes the vertex's cortical thickness, *m* and *n* are the number of points in *i* and *j*, respectively.

*In these formulas, i and j denote two brain regions; c(i,j) denotes the correlation between i and j*.

Like other papers, we selected cortical thickness as the single dimension feature to construct individual brain network. All methods employed an identical feature selection method after the constructions of each individual brain network and optimization of the parameters in SVM. The accuracy, sensitivity, specificity and area under receiver operating characteristic (ROC) curve (AUC) values of each method were calculated as evaluation metrics for the performance. The results are summarized in Tables 4, 5. It can be clearly observed that our method performed well compared with previous methods in the classification task. In particular, our method achieved an accuracy of 79.65% in distinguishing MCI patients from NC with a sensitivity of 78.82% and achieved an accuracy of 70.59% in distinguishing sMCI from pMCI with a sensitivity of 75.58%.

**Table 4:** Classification performance of different methods to distinguish MCI and NC.

**Method**	**Accuracy (%)**	**Sensitivity (%)**	**Specificity (%)**	**AUC**
**Our method using six dimensions**	**80.53**	**79.41**	**81.66**	**0.86**
**Our method using one dimension**	79.65	**78.82**	80.47	**0.84**
Kong's Method	77.88	74.12	81.66	**0.84**
Kim's Method	75.81	71.18	80.47	0.79
Dai's Method	76.70	73.53	79.88	0.82
Zheng's Method	**79.94**	76.47	**83.43**	**0.84**
Wee's Method	77.29	73.53	81.07	0.83

*One dimension denotes cortical thickness; six dimensions include cortical thickness, surface areas, gray matter volume, sulcal depth, metric distortion, and mean curvature. The lower bold values mean the best performance (accuracy, sensitivity, specificity and AUC) among different methods in one dimension situation. The upper bold values mean the best performance of our method in one and six dimension. AUC, area under the curve*.

**Table 5:** Classification performance of different methods to distinguish sMCI and pMCI.

**Method**	**Accuracy (%)**	**Sensitivity (%)**	**Specificity (%)**	**AUC**
**Our method using six dimensions**	**77.06**	**77.91**	**76.19**	**0.74**
**Our method using one dimension**	**70.59**	**75.58**	65.48	**0.73**
Kong's Method	65.89	67.44	64.29	0.67
Kim's Method	67.06	63.95	**70.24**	0.65
Dai's Method	63.53	70.93	55.95	0.64
Zheng's Method	67.65	63.95	71.43	0.68
Wee's Method	65.89	67.44	64.29	0.69

*One dimension denotes cortical thickness; six dimensions include cortical thickness, surface areas, gray matter volume, sulcal depth, metric distortion, and mean curvature. The lower bold values mean the best performance (accuracy, sensitivity, specificity and AUC) among different methods in one dimension situation. The upper bold values mean the best performance of our method in one and six dimension. AUC, area under curve*.

**Figure 4:**
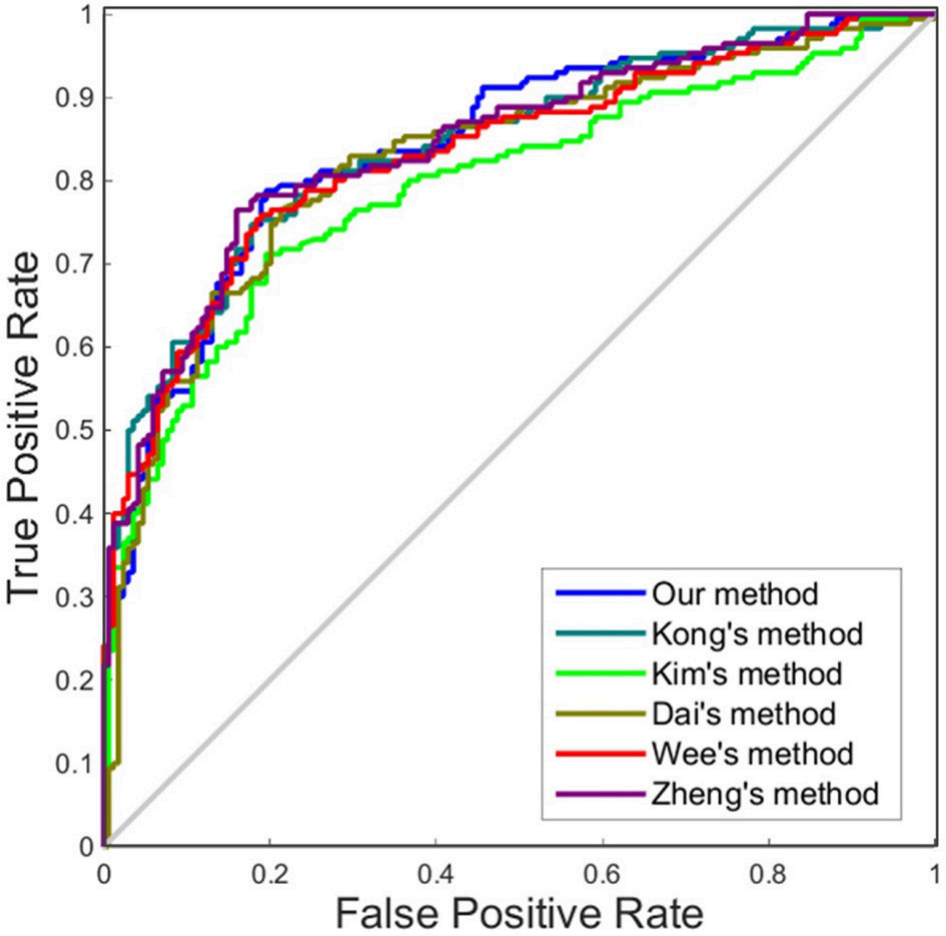
ROC curves of different methods using one dimension to distinguish MCI and NC. The different line colors represent different methods to construct individual morphological brain networks based on cortical thickness. ROC, receiver operating characteristic.

**Figure 5:**
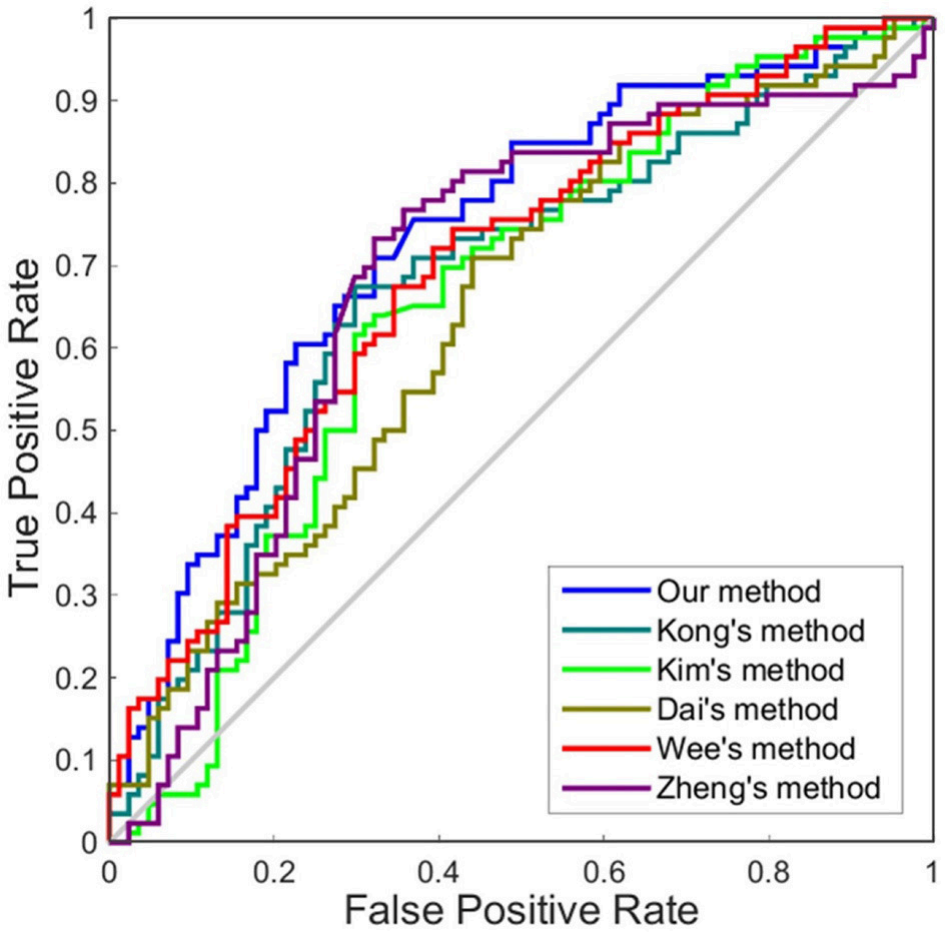
ROC curves of different methods using one dimension to distinguish sMCI and pMCI. The different line colors represent different methods to construct individual morphological brain networks based on cortical thickness. ROC, receiver operating characteristic.

Although accuracy is commonly used for an evaluation of classification, it may provide a biased description due to its dependency on the decision threshold selection in SVM. The ROC curve is shown to be a simple but completely empirical description of this decision threshold effect, indicating all possible combinations of the relative frequencies of the various kinds of correct and incorrect decisions. In ROC space, the (0, 1) point represents a perfect classifier (all samples are correctly predicted). Thus, the nearer a point is to the (0, 1) point (closer to the upper left corner), the better a classifier is (Prati et al., 2011). Figures 4, 5 show the ROC graphs of classification using different methods to construct individual brain networks, from which we can see that the ROC curve of our method is closer to the upper left corner than some conventional methods. In addition, a single measure of classification performance can be derived from the area under the ROC curve (AUC). A larger AUC indicates a better classifier. In Tables 4,5 the AUC for all methods are listed and it can be seen that our method achieved AUC scores of 0.84 for MCI vs. NC, and 0.73 for sMCI vs. pMCI, while most other methods slightly underperformed.

### Comparison of our method using one dimension and six dimensions in classification

**Figure 6:**
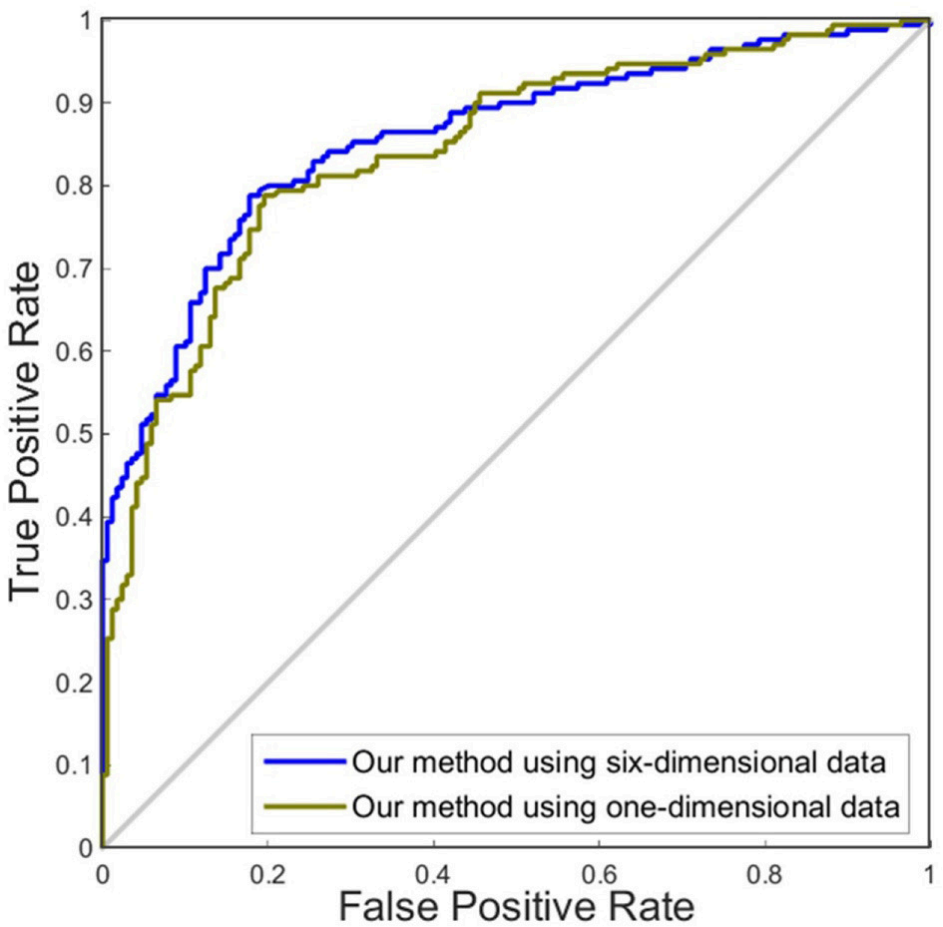
ROC curves of our method using different dimensions of original features to distinguish MCI and NC. The different line colors represent ROC curves of our methods of constructing individual morphological brain networks based on different dimensional features. ROC, receiver operating characteristic.

**Figure 7:**
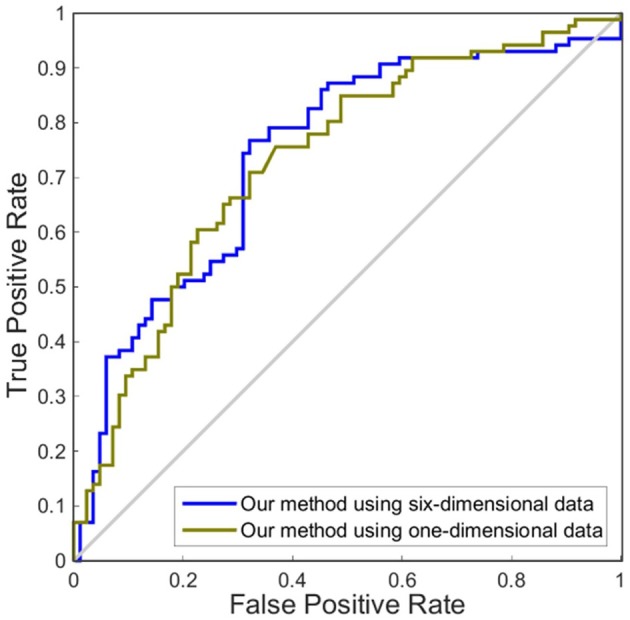
ROC curves of our method using different dimensions of original features to distinguish sMCI and pMCI. The different line colors represent ROC curves of our method of constructing individual morphological brain networks based on different dimensional features. ROC, receiver operating characteristic.

In this experiment, we compared the performance of the proposed method by using one dimension and six dimensions. We used cortical thickness as the single dimension and used cortical thickness, surface areas, gray matter volume, sulcal depth, metric distortion and mean curvature as the six dimensions. Tables 4,5 show that our method of applying six dimensions outperforms the one only using a single cortical thickness feature, which achieved 80.53% and 77.06% for accuracy in distinguishing MCI from NC and distinguishing sMCI from pMCI, respectively. The ROC graphs in Figures 6,7 illustrate the classification performance based on brain networks that were constructed using one dimension and six dimensions. We also list the AUC score in Tables 4,5. It can be noticed that compared with the univariate situation, individual brain network construction based on multivariate performs better in classification with an AUC score of 0.86 and 0.74, respectively.

### Most discriminative features of individual brain networks

**Figure 8:**
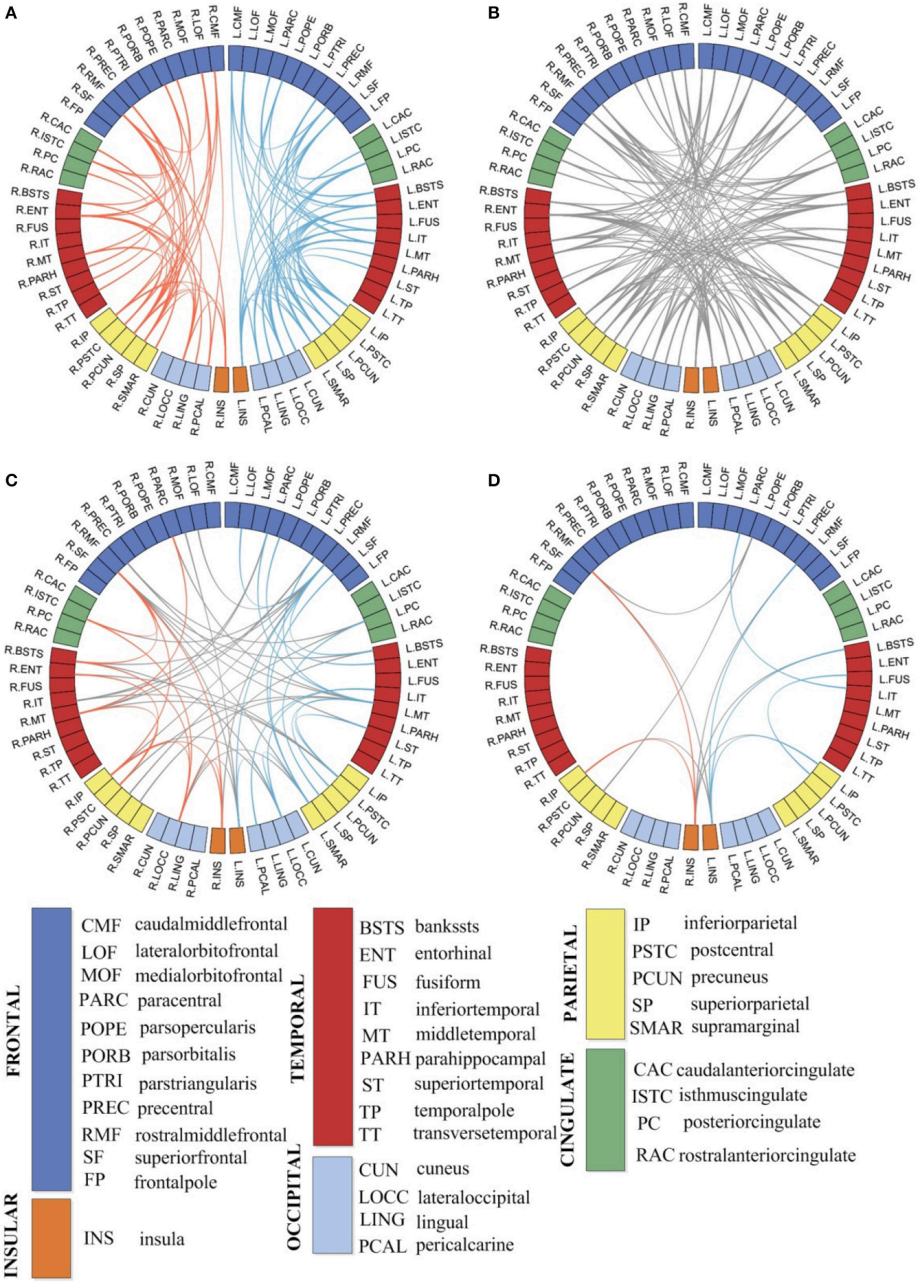
The most discriminative edges of individual morphological brain networks in classification **(A,B)** and the significant correlation of the most discriminative edges with MMSE **(C)** and CDR **(D)** scores. L, left hemisphere; R, right hemisphere; the different colors of the blocks represent ROIs in different areas of the cortical surface. The blue lines represent the discriminative edges in the left hemisphere; the red lines represent the discriminative edges in the right hemisphere. The gray lines represent the discriminative edges between the left and right hemisphere.

The most discriminative features demonstrate the edges selected in each time of cross-validation for classification based on multivariate connectivity. Here, we selected the most discriminative features under the best condition. In Figure 8, the blocks of the circle represent ROIs. As shown in Figures 8A,B the most discriminative edges connected most ROIs in the brain.

Based on the selected edges, pairs of regions that contribute to classification are not only within the same hemisphere and the same lobe but also across different hemispheres and lobes, which indicates the abnormalities caused by MCI involve the entire brain rather than certain areas. The number of discriminative edges that connect the two hemispheres was 115. Conversely, the number of discriminative edges that are the connections within a single hemisphere was relatively low, with quantities of 64 and 43 for the left and right hemisphere, respectively. We correlated the most discriminative edges with MMSE and CDR scores. In Figures 8C,D, the selected edges that were significant correlated (*p* < 0.05) with MMSE and CDR are shown. As seen, these edges are predominately in the frontal, temporal, parietal, and insula parts.

## Discussion

In the present study, we introduced a new method to construct individual morphological brain network. The combination of inter-regional Euclidean distance and intra-regional Euclidean distance was used to quantify the inter-regional relations. Through the small-world configurations analysis, our method confirmed the existence of small world property. In addition, as listed in Table 2, the population-based morphological brain networks and functional networks exhibit smaller results than our results in most small world configurations, which may suggest that the individual morphological brain networks demonstrate a stronger integration and segregation because the inter-individual variability is highly reserved (Kanai and Rees, 2011). Hubs such as left and right frontal pole and right rostral anterior cingulate have been reported in previous studies (Hagmann et al., 2008; Van den Heuvel and Sporns, 2013). The ICC was used to estimate the reproducibility of graph theoretical measures. The results indicated that the reliability of our method performed well in accordance with previous studies. In addition, compared with other conventional methods, which average the vertices within ROIs, our method improves the classification performance in univariate situation. Here, we explained the rationality of our method from two aspects. (1) In previous studies, the individual morphological brain networks were mostly constructed based on the average value of morphological features within the ROI. However, the abnormal region for pathology might be only a fraction of the defined ROI and the abnormal change of brain region may be ignored by taking the average, which potentially reduces the discriminative power. In our proposed method, we directly used the morphological features of vertices to retain more detailed information. The results of Kong's method (Kong et al., 2014) and Zheng's method (Zheng et al., 2015) in Table 4 also demonstrated the importance of detailed information. (2) In previous studies, the morphological distribution within an ROI was not considered, which may influence the strength of edges between ROIs. In our method, the dissimilarity connectivity was the combination of inter-regional Euclidean distance and intra-regional Euclidean distance, while previous methods only considered the relation between two ROIs.

An inherent advantage of our method is that it can be applied to multi-dimensional situations. In previous studies, researchers have found the small-world properties were disrupted for brain networks that were constructed based on cortical thickness in MCI patients (Zhou and Lui, 2013), and the brain network based on the surface area can reveal topological properties of the networks resulting from the concurrent changes between different anatomical regions (Sanabriadiaz et al., 2010). The sulcal depth, curvature, and metric distortion related to cortical folding vary and could be more suitable descriptors for finding the anatomical-axonal and morphological connectivity correlation (Van Essen, 1997). Previous studies have reported that brain networks based on both the volumetric measures and geometric measures showed significant differences in graphical properties between aMCI and NC (Li et al., 2016). These results may suggest that brain network construction based on multiple features is beneficial to the diagnosis and analysis of neurological diseases. However, most previous approaches (Dai et al., 2013; Wee et al., 2013; Kong et al., 2014; Zheng et al., 2015; Kim et al., 2016) that constructed individual brain networks only considered one morphological feature (e.g., cortical thickness or gray matter volume) between two brain regions. The first paper involved in building morphological brain networks based on multiple morphological features demonstrated that multiple morphometric features can be applied to form a rational reproducible individual-based morphological brain network (Li et al., 2017), but it averaged the morphological features within each ROI, such as the mean cortical thickness, which may neglect some detailed information. In our method, every vertex's different kinds of cortical features within each ROI were considered and the relations between brain regions were determined based on these features. In this paper, the multiple morphological features including cortical thickness, surface areas, gray matter volume, sulcal depth, metric distortion and mean curvature as well as the cortical thickness as a single feature were used for individual brain network construction. The results show (Tables 4,5) that the brain network constructed from the combination of morphological features outperforms the one only considering cortical thickness. The resulting high AUC value proves the excellent classification power and generalizability of our proposed method on an unseen data set, as well as the ability to construct an accurate and credible individual morphological brain network. Moreover, the classification performance of our method in a multivariate situation revealed the existence of useful information within these morphological features. The abnormal connectivity across various regions can be located within different morphological features, which greatly benefits the detection of neurological diseases.

An interesting finding shown in Figures 8A,B is that the majority of the selected correlative features in the MCI and NC classification task are the edges connecting the left and right hemisphere. This might suggest that the most significant differences between MCI subjects and health subjects are changes in the connections between the left and the right hemisphere. The connection alterations caused by MCI pathological attacks are not restricted to certain brain areas but are widely spread over the whole brain. What's more, the most discriminative edges connecting the regions in our study are consistent with previous publications, such as the lingual gyrus, postcentral gyrus, middle temporal gyrus, pars opercularis, and superior frontal sulcus (Li et al., 2014; Wei et al., 2016). Previous studies have found that subjects with MCI have abnormal network patterns in the lingual gyrus and middle temporal gyrus (Yao et al., 2010). He et al. (2008) demonstrated an abnormal correlation between the bilateral postcentral gyrus in AD. From Figures 8C,D we can see the selected edges are predominately connected to the regions of the frontal, temporal, parietal, and insula parts. These regions have been reported that retain more hubs which are considered to be the substrates of human cognition and consciousness (Yao et al., 2010). In addition, some regions are associated with changes in different morphological features in MCI subjects, such as the middle frontal gyrus with cortical thickness, the postcentral gyrus with metric distortion, the pars opercularis with mean curvature, the lingual gyrus with surface area, and the superior frontal sulcus with sulcal depth (Li et al., 2014). In conclusion, our results suggest that changes in the cortical regions may be associated with mechanisms underlying the conversion of MCI to AD, and the changes were displayed in multiple morphological features. These findings illustrate the potential application of our proposed method.

There are still some limitations in this study. First, the selection of the brain atlas could affect the organization of the individual brain network (Wang et al., 2016). In the future, it is important to validate our proposed method in different atlases. Second, in the current study, we combined multiple morphological features to construct the individual network, and we validated the effectiveness of our method. However, it is noticeable that the physiological explanation of this network is difficult. Third, a recent study (Seidlitz et al., 2017) proposed an individual brain network method by estimating the inter-regional correlation based on multiple macro- and micro-structural multimodal MR variables. And this network could capture cellular, molecular and functional features of the brain and even predict inter-individual differences in cognition. In future, it would be interesting to employ multiple morphometric parameters measured using multimodal MRI. Last, each feature type had its distinct contribution when discriminating between two groups. In the future, we may first select the most discriminant features and then construct the individual network, which could improve its classification performance.

## Author contributions

KY, XW, and SL designed the experiments. XL assembled the data. KY performed the experiments and prepared the manuscript. XW, QL, and XZ helped in manuscript writing. SL was in charge of manuscript verification. All authors reviewed the manuscript.

### Conflict of interest statement

The authors declare that the research was conducted in the absence of any commercial or financial relationships that could be construed as a potential conflict of interest.
